# Supplementary material for: Mechanisms of Individual and Simultaneous Adsorption of Antibiotics and Dyes onto Halloysite Nanoclay and Regeneration of Saturated Adsorbent via Cold Plasma Bubbling
Source: Nanomaterials (Basel). 2023 Jan 13;13(2):341. doi: 10.3390/nano13020341 (PMC9862438; doi:10.3390/nano13020341)
Supplement: Supplementary file 1 [file nanomaterials-13-00341-s001.zip › nanomaterials-2138938-supplementary.pdf]

nanomaterials

Article

Mechanisms of Individual and Simultaneous Adsorption of Antibiotics and Dyes onto Halloysite Nanoclay and Regeneration of Saturated Adsorbent via Cold Plasma Bubbling

Stefania Giannoulia [1,2], Irene-Eva Triantaphyllidou [1], Athanasia G. Tekerlekopoulou [2] and Christos A. Aggelopoulos [1,*]

[1] Laboratory of Cold Plasma and Advanced Techniques for Improving Environmental Systems, Institute of Chemical Engineering Sciences, Foundation for Research and Technology Hellas (FORTH/ICE-HT), 26504 Patras, Greece
[2] Department of Sustainable Agriculture, University of Patras, 2 G. Seferi St., 30100 Agrinio, Greece
* Correspondence: caggelop@iceht.forth.gr; Tel.: +30-2610965205

S1 Main characteristics of methylene blue, enrofloxacin and halloysite nanoclay used in this study

The antibiotic enrofloxacin (ENRO, $C_{19}H_{22}FN_3O_3$, M.W.= 359.39 g/mol) and methylene blue (MB, $C_{16}H_{18}ClN_3S \bullet 2H_2O$, M.W. = 373.90 g/mol) are characteristic molecules in the class of antibiotics and dyes, respectively. Enrofloxacin (ENR) is a fluoroquinolone antibiotic being widely used in veterinary medicine. It has antibacterial activity against a broad spectrum of Gram-negative and Gram-positive bacteria, effective against a plethora of diseases. Methylene blue is a salt used as a dye and medication, being classified as a thiazine dye. Halloysite ($Al_2(OH)_4Si_2O_5 \bullet nH_2O$) is a natural aluminosilicate nano-clay mineral (HNC), that has tubular structure and usually is occurs through intermix with other clay-minerals (e.g. kaolinite, montmorillonite, etc.). Each halloysite layer consists of tetrahedral (Si–O) and octahedral (Al–OH) sheets and one alumina octahedron sheet. Halloysite nanotubes have a wall thickness of 10–15 atomic aluminosilicates sheets, outer diameter of 50–60 nm, inner diameter 12–15 nm and length 0.5–10 μm. The outer surface is usually covered by the tetrahedral Si–O and the inner by the octahedral Al–OH and hence the two surfaces bear opposite charge.

The molecular structure and the main physicochemical properties of enrofloxacin, methylene blue and halloysite nanoclay are shown in Table S1. Moreover, for the High-Resolution Transmission Electron Microscopy (HR-TEM) images for HNC, TEM JEOL 2100 microscope (200 kV) was used (Figure S1).

Table S1. Molecular structure and physicochemical properties of MB and ENRO.

	Methylene blue	Enrofloxacin	Halloysite nanoclay
CAS No.	122965-43-9	93106-60-6	1332-58-7
Formula	$C_{16}H_{18}ClN_3S$	$C_{19}H_{22}FN_3O_3$	$Al_2(OH)_4Si_2O_5 \cdot 2H_2O$
Molecular weight	373.90 g/mol	359.39 g/mol	294.19 g/mol
Melting point	100-110 °C	225 °C	-

Solubility in water	43.6 mg/mL	150 µg/mL	-
Relative density	-	-	2.53 g/cm^3
Molecular structure	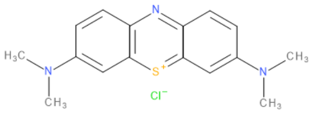	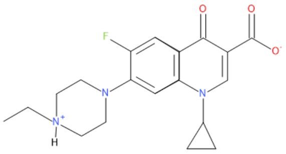	

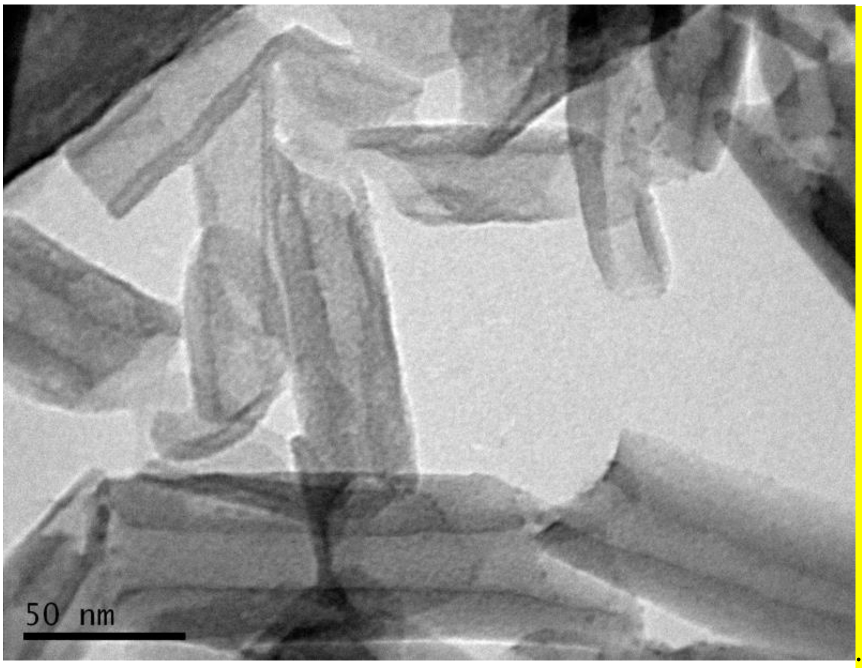

Figure S1. High-Resolution Transmission Electron Microscopy (HR-TEM) image for HNC.

S2 Point of zero charge

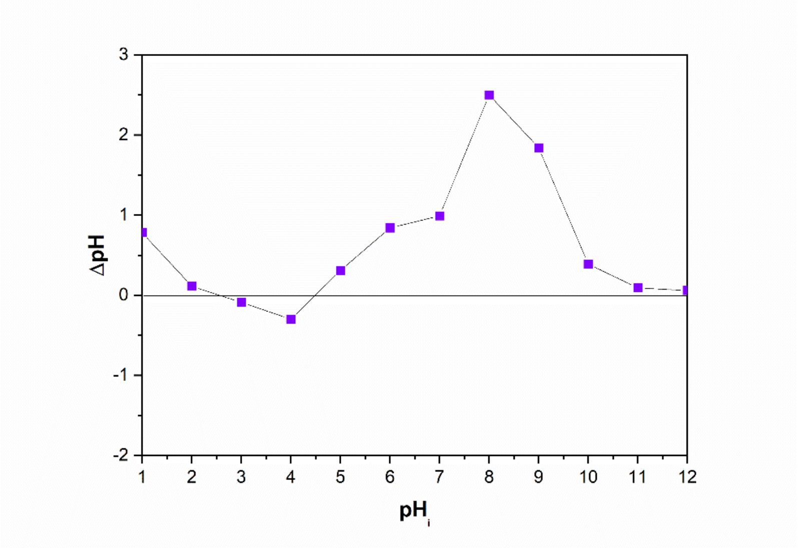

Figure S2. ΔpH versus pH$_i$ for HNC.

S3 ATR/FTIR

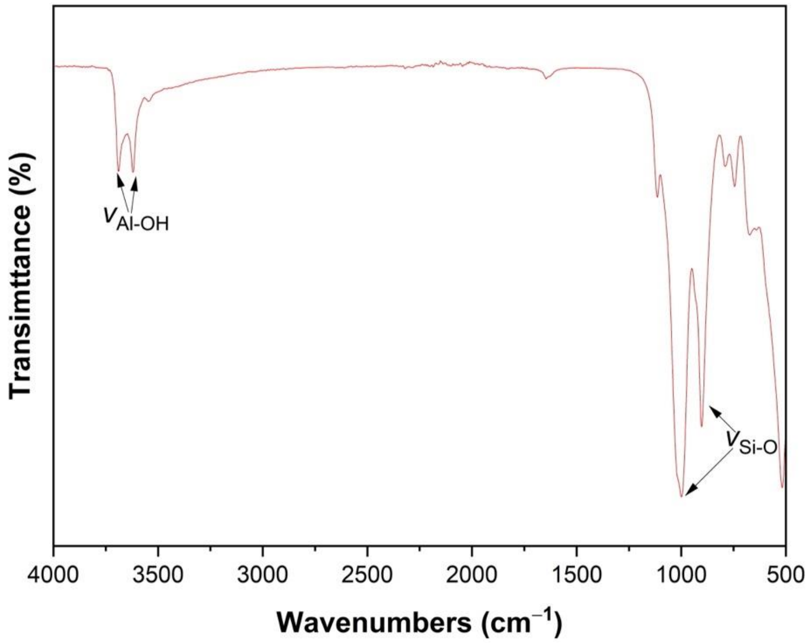

Figure S3. The ATR/FTIR spectrum of halloysite in the spectral region 500–4000 cm^{-1}.

S4 Kinetic data analysis for the single system

The pseudo first order model for ENRO and MB:

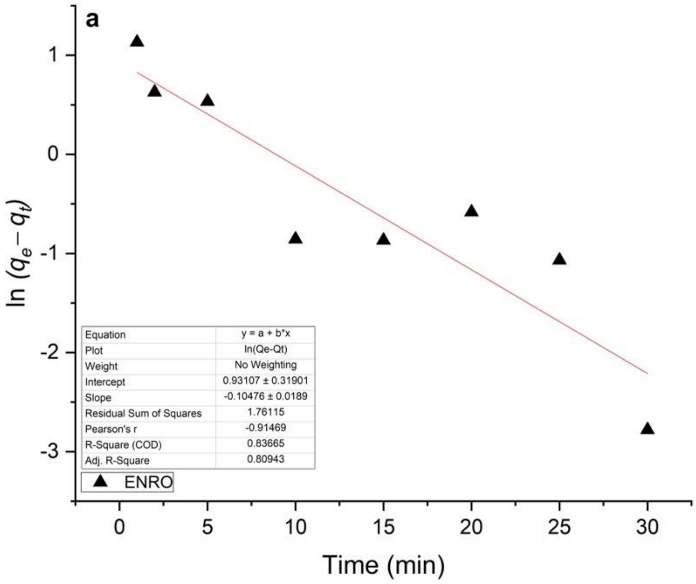

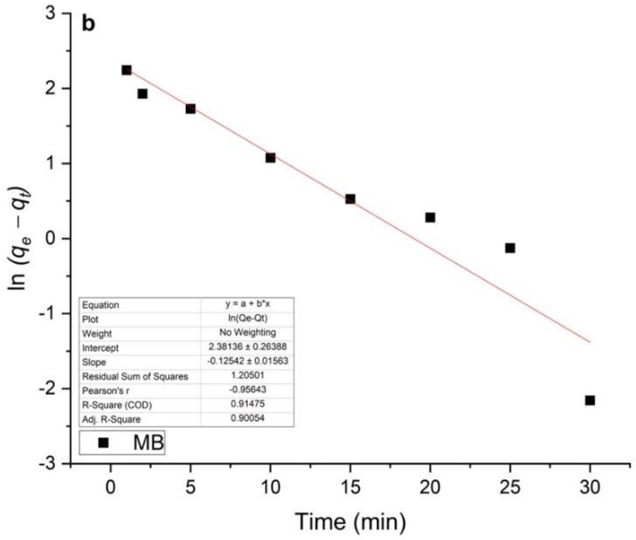

Figure S4. Pseudo-first order reaction kinetics for the adsorption of ENRO (**a**) and MB (**b**) on HNC in the single system.

The pseudo second order model for ENRO and MB:

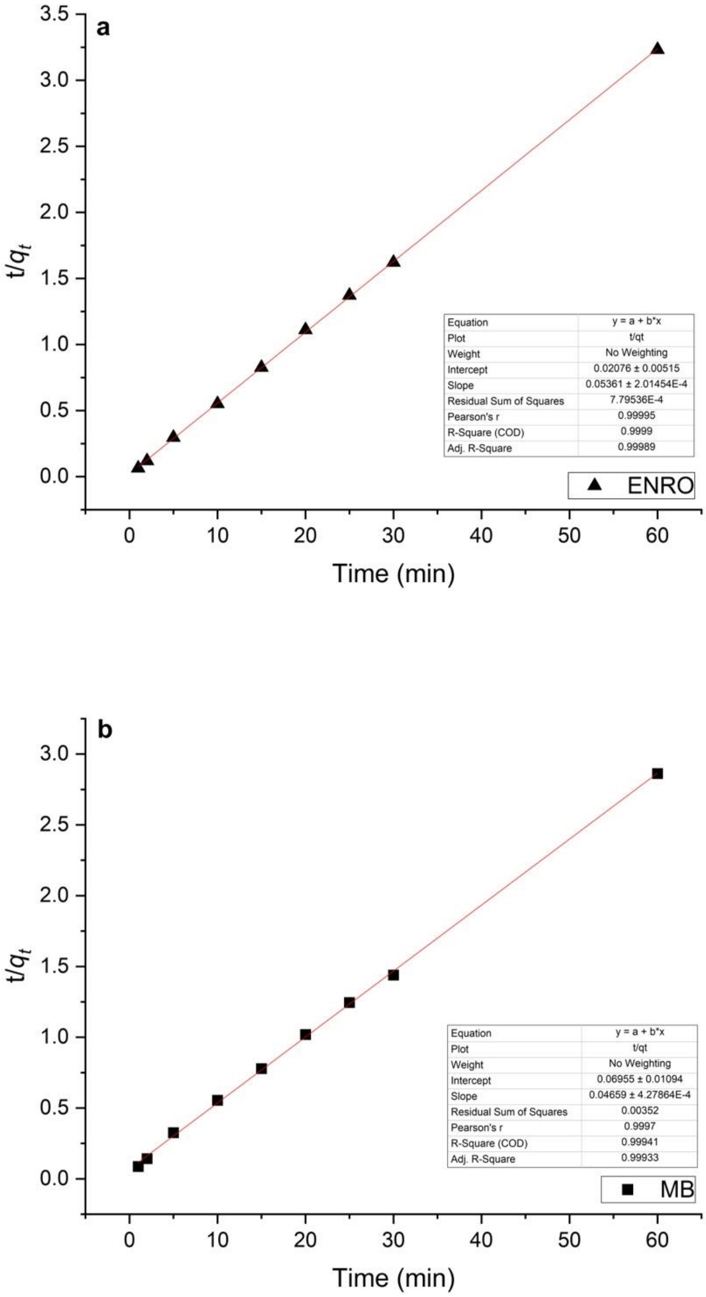

Figure S5. Pseudo-second order reaction kinetics for the adsorption of ENRO (**a**) and MB (**b**) on HNC in the single system.

The Elovich model for ENRO and MB:

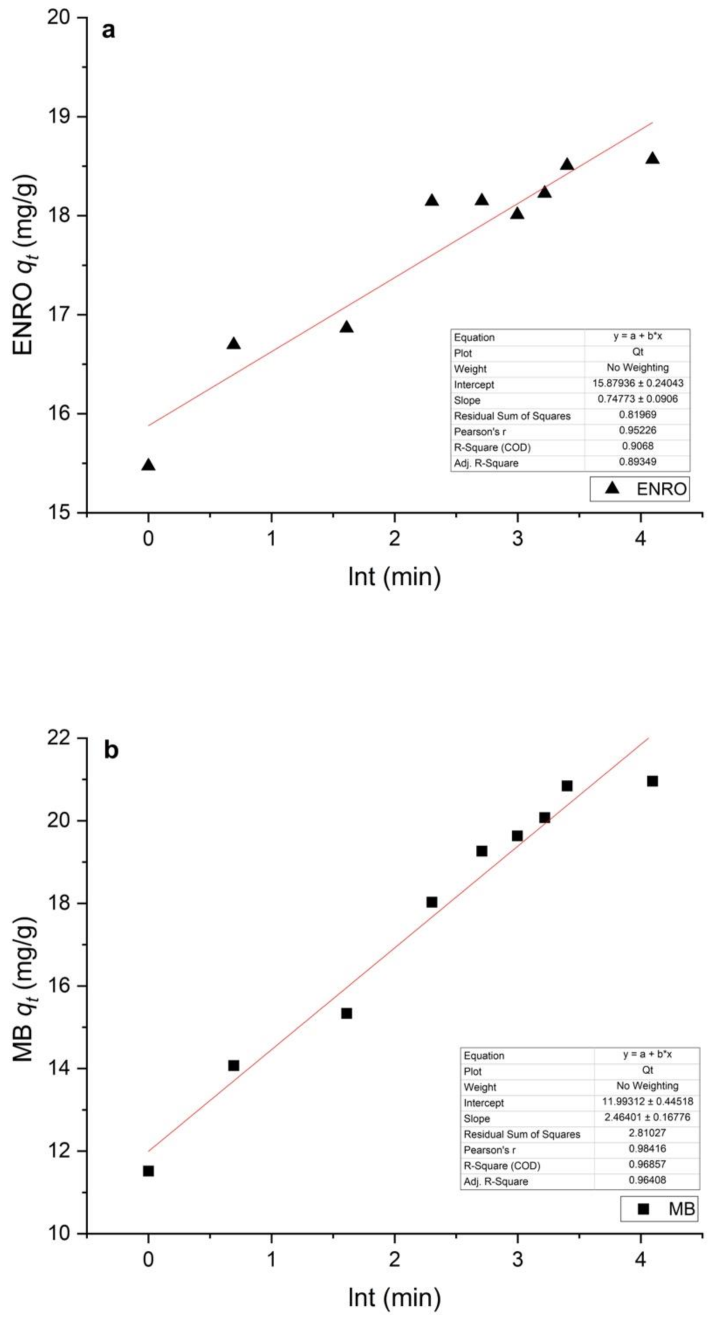

Figure S6. Elovich kinetic parameter for the adsorption of ENRO (**a**) and MB (**b**) on HNC in the single system.

The Weber–Morris intra-particle diffusion model for ENRO and MB:

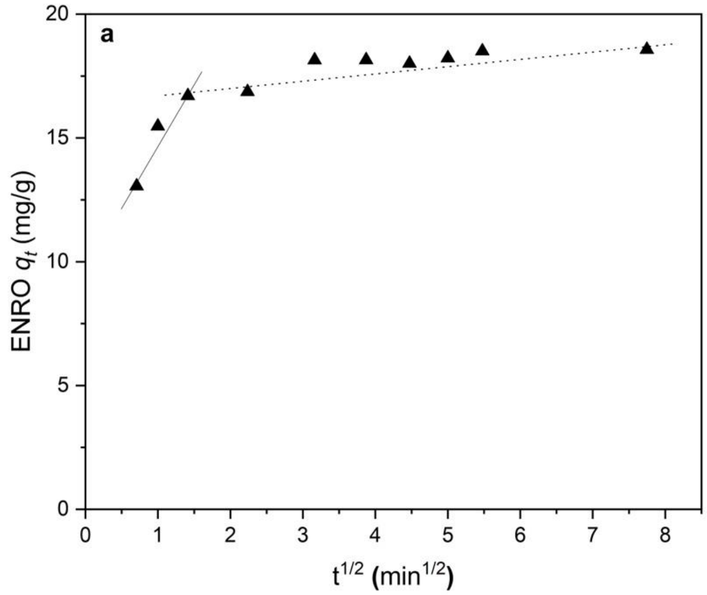

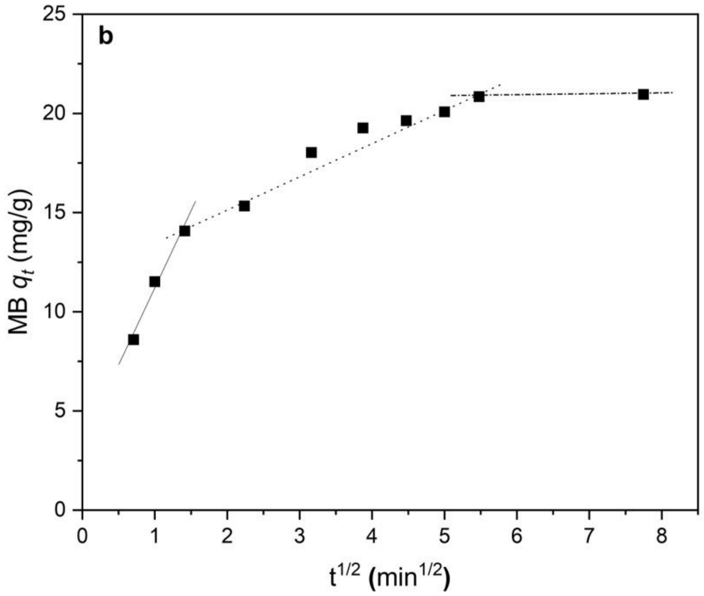

Figure S7. Intra-particle diffusion model curves for the adsorption of ENRO (**a**) and MB (**b**) on HNC, for the single system.

S5 Kinetic data analysis for the binary system

The pseudo first order model for ENRO and MB:

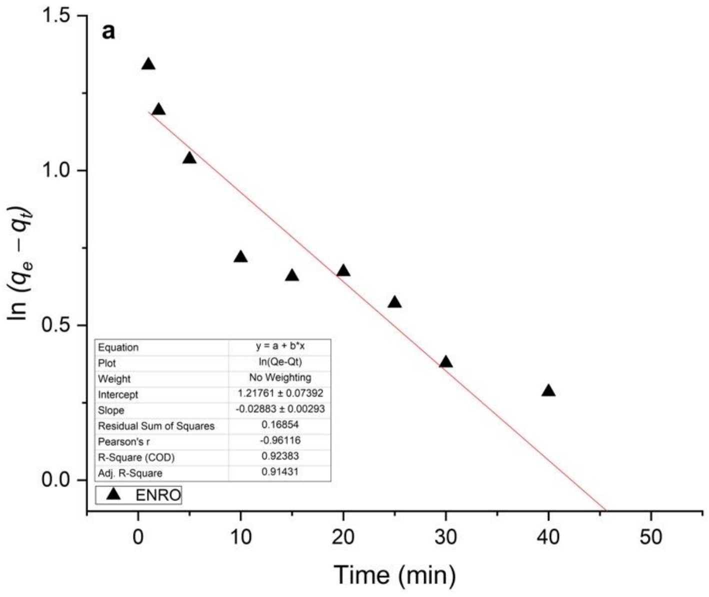

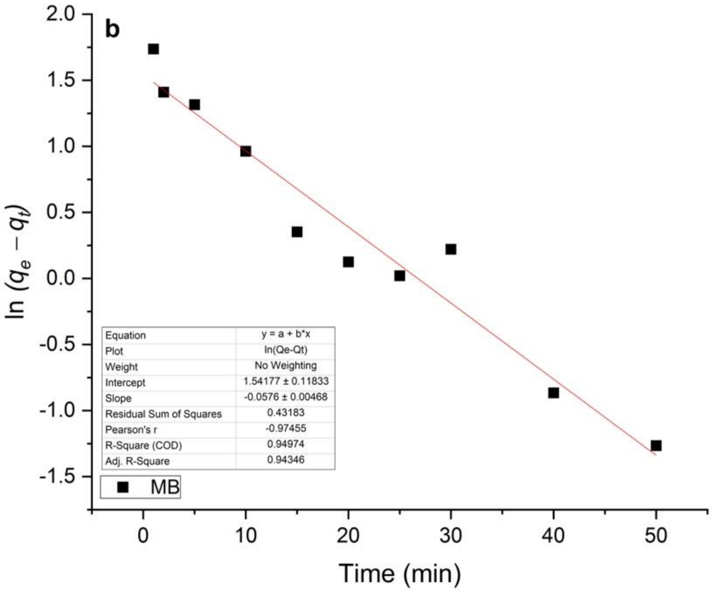

Figure S8. Pseudo-first order reaction kinetics for the adsorption of ENRO (**a**) and MB (**b**) on HNC in the binary system.

The pseudo second order model for ENRO and MB:

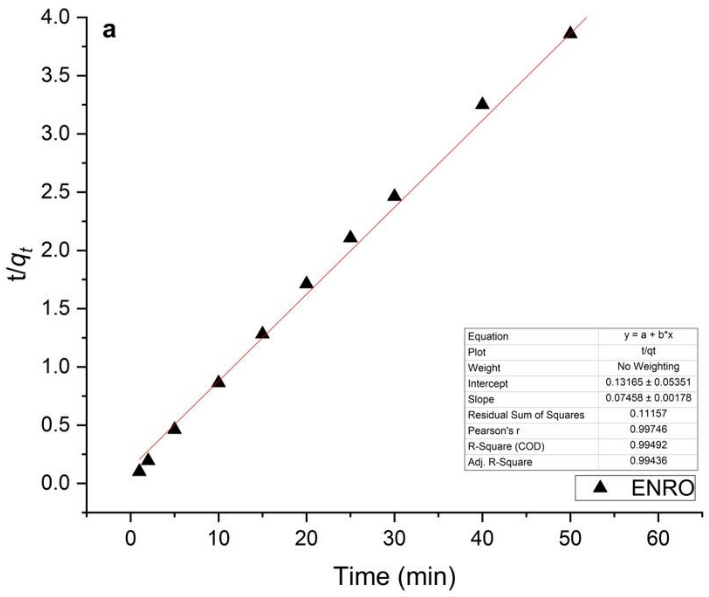

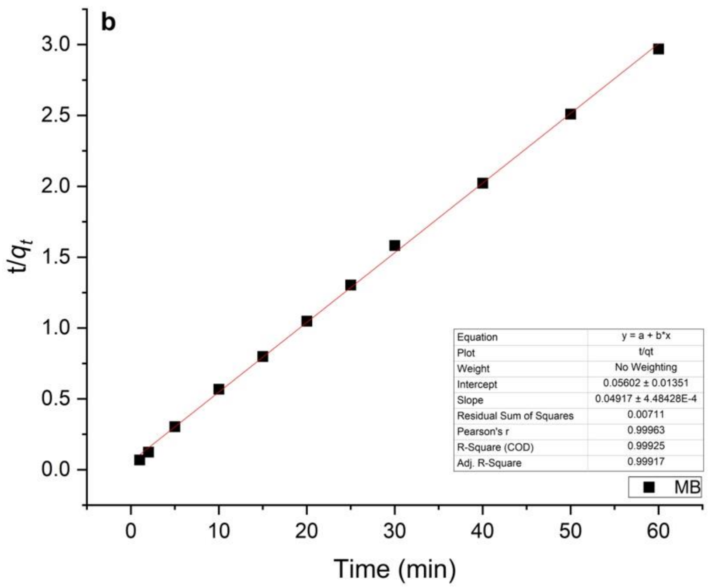

Figure S9. Pseudo-second order reaction kinetics for the adsorption of ENRO (**a**) and MB (**b**) on HNC in the binary system.

The Elovich model for ENRO and MB:

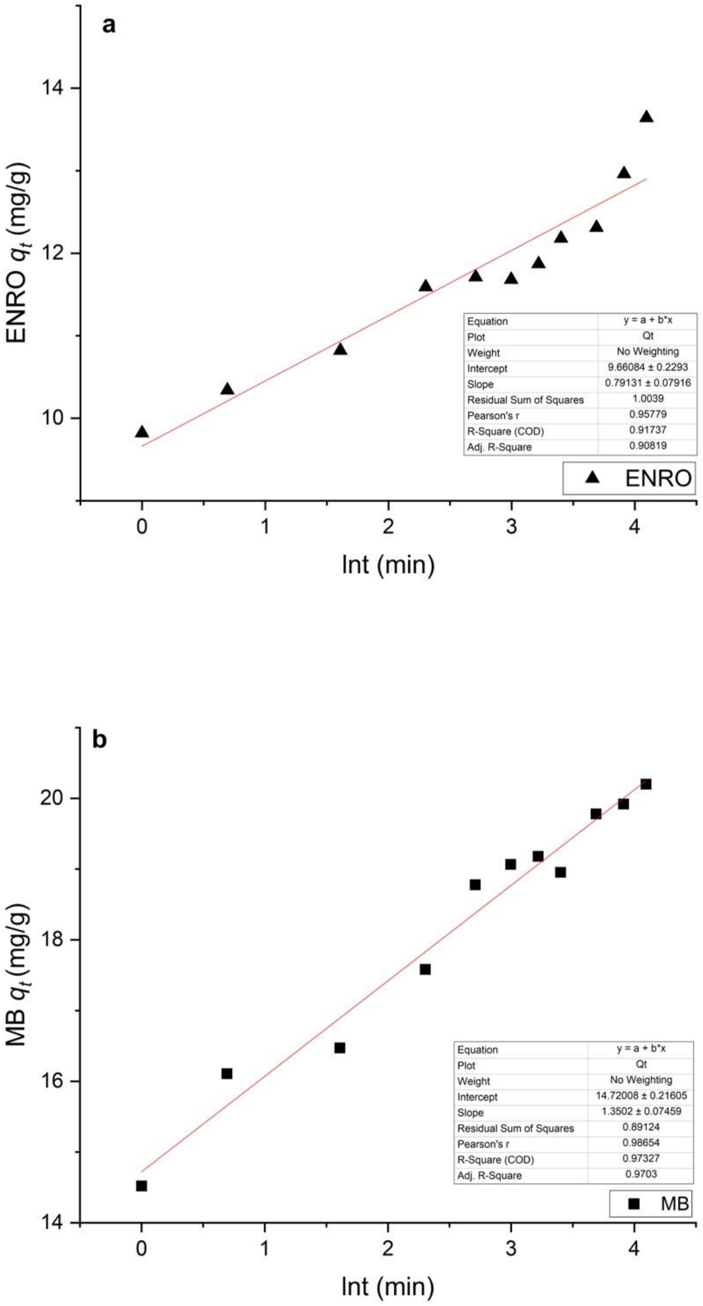

Figure S10. Elovich kinetic parameter for the adsorption of ENRO (**a**) and MB (**b**) on HNC in the binary system.

The Weber–Morris intra-particle diffusion model for ENRO and MB:

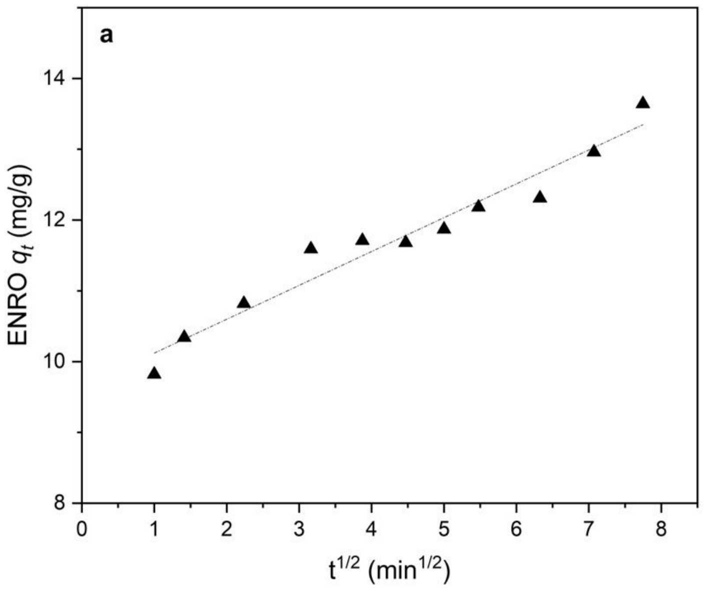

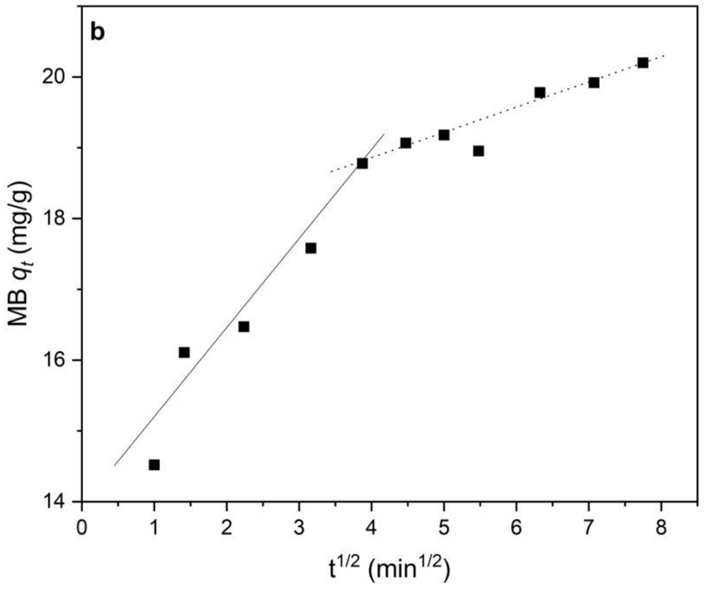

Figure S11. Intra-particle diffusion model curves for the adsorption of ENRO (**a**) and MB (**b**) on HNC, for the binary system.
